# Change trajectory of dyadic coping and subjective well-being in patients with malignant bone tumors based on cross-lagged panel model and latent growth model: a longitudinal study

**DOI:** 10.3389/fpsyg.2025.1563458

**Published:** 2025-08-15

**Authors:** Rui Li, Chun-yan Zhang, Yue Wu, Yao Wang, Yi-fan Li, Xiao-juan Fan, Hong Song

**Affiliations:** ^1^Department of Orthopaedic, The Second Affiliated Hospital of Xuzhou Medical University, Xuzhou, Jiangsu, China; ^2^Department of Neurosurgery, The Second Affiliated Hospital of Xuzhou Medical University, Xuzhou, Jiangsu, China; ^3^Department of Nursing, The Second Affiliated Hospital of Xuzhou Medical University, Xuzhou, Jiangsu, China

**Keywords:** malignant bone tumors, dyadic coping, subjective well-being, latent variable growth model, cross-lagged model, nursing care

## Abstract

**Background:**

Malignant bone tumors can result in physical disability, which has a significant impact on the quality of patients’ survival. Additionally, patients often experience high levels of psychological distress. The subjective well-being of patients with bone tumors is low, and this low level of well-being is the direct cause of the accumulation of negative emotions and misanthropy in patients.

**Objective:**

The objective of this study is to examine the developmental trajectory of dyadic coping and subjective well-being in patients with malignant bone tumors, and to investigate the predictive relationship between the two. Furthermore, this study aims to provide a theoretical basis for improving the subjective well-being of patients with malignant bone tumors.

**Methods:**

A total of 265 patients with malignant bone tumors who were hospitalized in the Department of Orthopedics of the Second Affiliated Hospital of Xuzhou Medical University in Xuzhou City, Jiangsu Province from October 2021 to April 2024 were selected as the research subjects. Their dyadic coping and subjective well-being were tracked and examined at three time points: T1 (at the time of diagnosis), T2 (1 month after diagnosis), and T3 (3 months after diagnosis). The data were analyzed using a cross-lagged model and a latent variable growth model.

**Results:**

The cross-lagged modeling revealed that, on average, dyadic coping levels exhibited a significant and positive predictive relationship with subjective well-being at the subsequent node. Similarly, subjective well-being levels demonstrated a significant and positive predictive relationship with dyadic coping levels at the subsequent node. The latent variable growth model demonstrated an upward trajectory in dyadic coping (S = 0.228, *p* = 0.047) and an upward trajectory in subjective well-being (S = 0.109, *p* = 0.212) in patients with malignant bone tumors from T1 to T3. Furthermore, dyadic coping exhibited a positive correlation with well-being at the initial well-being (*r* = 0.533, *p* < 0.001). Furthermore, there was a negative interaction between the rate of progression prediction and the initial level of subjective well-being (*β* = −0.480, *p* = 0.008). Additionally, the initial level of subjective well-being and the developmental rate negatively predicted each other (*β* = −0.749, *p* = 0.005). Notably, the initial level of subjective well-being was able to positively predict the developmental rate of dyadic coping (*β* = 0.294, *p* < 0.001).

**Conclusion:**

The results demonstrated a notable increase in dyadic coping and subjective well-being in patients with malignant bone tumors from the time of diagnosis to 3 months post-diagnosis. Furthermore, there was a discernible correlation between dyadic coping and subjective well-being in patients with malignant bone tumors.

## Introduction

Malignant bone tumors are neoplastic lesions occurring in the bone and its appendant tissues. Their incidence is increasing year by year, with a higher incidence in men than in women (Huang et al., 2023). The 5-year survival rate is less than 30% (Brisset et al., 2015). Data shows that there were 28,000 new cases of malignant bone tumors in China in 2023 (Luo et al., 2021). Subjective Well-Being (SWB) encompasses an individual’s cognitive appraisal of life satisfaction and affective experiences of positive versus negative emotions, reflecting a personalized evaluation of overall quality of life (Diener, 1984). Malignant bone tumors are the primary cause of limb disability, which significantly impacts the quality of life and psychological well-being of patients (Wang et al., 2018). The quality of life is the foundation for the experience of subjective well-being (Wiki et al., 2023; Topp et al., 2015), and patients with bone tumors may exhibit diminished levels of subjective well-being. A low level of well-being directly causes the accumulation of negative emotions and anhedonia in patients (Lovero et al., 2023; Tanriverdi et al., 2024).

Dyadic coping refers to the dyadic process in which partners jointly respond to stressful events through coordinated communication, collaborative problem-solving, and mutual emotional support (Rentscher, 2019). It has been demonstrated that effective dyadic coping can mitigate the negative emotions experienced by patients, enhance their cooperation and quality of life, and improve their prognosis (Cai et al., 2021; Staff et al., 2017; Margola et al., 2018). It is therefore important to gain an understanding of the trajectory of subjective well-being and its interaction with dyadic coping in patients with bone tumors after diagnosis, in order to provide proactive interventions for the high prevalence of low levels of subjective well-being. This will contribute to maintaining patient safety and improving prognosis.

However, domestic and international studies on dyadic coping and subjective well-being are limited to cross-sectional studies (Lameiras et al., 2018). Furthermore, there is a paucity of research examining the developmental trajectories of dyadic coping and subjective well-being in patients with bone tumors, as well as their dynamic interrelationships. Therefore, we propose the following hypotheses:

*H*1: The dyadic coping and subjective well-being of patients with malignant bone tumors exhibit dynamic changes over time.

*H*2: Dyadic coping predicts subjective well-being in patients with malignant bone tumors.

*H*3: Subjective well-being predicts dyadic coping in patients with malignant bone tumors.

*H*4: The initial level and rate of change in dyadic coping and subjective well-being demonstrate bidirectional predictive relationships in patients with malignant bone tumors.

This study employs a longitudinal design and utilizes cross-lagged and latent variable growth models to examine the trajectories of dyadic coping and subjective well-being changes, as well as the inter-predictive relationships among patients with bone tumors after diagnosis. The aim is to provide a theoretical basis for clinical staff to enhance the level of subjective well-being in patients with bone tumors.

## Subjects and methods

### Subjects

A total of 265 patients with malignant bone tumors admitted to the Department of Orthopedics of the Second Affiliated Hospital of Xuzhou Medical University between October 2021 and April 2024 were selected as study subjects. The following criteria were used to determine which patients were eligible for inclusion in the study: (1) The patient fulfills the diagnostic criteria established in the 2020 World Health Organization Classification of Soft Tissue and Bone Tumors (Choi and Ro, 2021; Anderson and Doyle, 2021). Based on comprehensive clinical evaluation, imaging studies, and histopathological examination, the diagnosis confirms a primary malignant bone tumor; (2) Patients were aged 18 years or older, married, and with a spouse aged 18 years or older; (3) Patients demonstrated a basic understanding and communication ability, and all signed the informed consent form. The following criteria were used to exclude participants from the study: (1) A history of psychiatric and psychological diseases; (2) Metastatic malignant bone tumor, currently receiving psychotropic drugs, psychotherapy. Additionally, a number of patients died or withdrew from the study for various reasons.

## Methods

### Sample size calculation

According to the sample size requirement of the latent variable growth model (Liu et al., 2014), at least 200 patients were needed, and considering the possible high dropout rate of 3 measurements, the dropout rate was set at 15%, so the minimum sample size for this study was set at *n* = 200/(1–15%) = 235 cases.

### Investigation tools

1. The initial phase of the study involved the administration of a self-report questionnaire to obtain basic demographic information, including the participants’ age, gender, marital status, monthly income, educational background, and occupation.
2. The Dyadic Coping Inventory (DCI), originally developed by Bodenmann et al. and later adapted for the Chinese population by Xu et al., is a 37-item scale assessing six dimensions of dyadic coping: stress communication (4 items), supportive coping (10 items), empowering coping (5 items), negative coping (8 items), joint coping (8 items), and coping quality evaluation (2 items). The instrument uses a 5-point Likert scale, with total scores ranging from 35 to 175. Scores are interpreted as follows: below-average dyadic coping (<111), normal dyadic coping (111–145), and above-average dyadic coping (>145), where higher scores indicate more positive dyadic coping patterns in couples (Xu et al., 2016). The Cronbach’s alpha coefficient for this scale in the present study was 0.810.
3. The Chinese version of the Subjective Well-Being Schedule, adapted by Duan in 1996 to reflect Chinese cultural characteristics, is an 18-item scale organized into six dimensions that employs three distinct rating systems: a 5-point scale for items 2, 5, 6, and 7; a 10-point scale for items 15–18; and a 6-point scale for the remaining items, yielding a total possible score of 120 points that categorizes well-being levels as low (0–48), moderate (49–72), or high (73–120), with higher scores indicating greater subjective well-being. This version maintains the original scale’s psychometric properties while incorporating culturally appropriate modifications to ensure valid assessment within the Chinese population context (Duan, 1996). The Cronbach’s alpha coefficient for this scale in the present study was 0.862.

### Data collection method

The questionnaires were administered in person in the orthopedic wards following the requisite consent procedures with the hospital and patients. The researchers screened potential participants according to the inclusion criteria. Baseline demographic data were collected during patients’ initial diagnosis and hospitalization. The Dyadic Coping Inventory and Subjective Well-Being Schedule were administered at three time points: T1 (during hospitalization at initial diagnosis), T2 (1 month post-diagnosis), and T3 (3 months post-diagnosis). While the T1 questionnaires were completed during the hospital stay, the T2 and T3 follow-up assessments were conducted via telephone after patients’ discharge. To guarantee the confidentiality of the participants, the survey was conducted in a secure setting and the participants were informed that the data would be used exclusively for the purposes of this study. The questionnaire was completed anonymously, without the disclosure of any personal information or the exploration of individual factors. This study employed longitudinal tracking through the Wenjuanxing platform, with three-phase data matching achieved using unique identification codes (the last four digits of participants’ mobile phone numbers). The T1 assessment was completed via QR code scanning during initial questionnaire distribution. Follow-up assessments at T2 (±7 days) and T3 (±7 days) were conducted through scheduled WeChat push notifications. Patients with low literacy and dyslexia were invited to repeat the scale entries, which they then completed independently. To ensure the reliability of the questionnaire and accurately reflect patients’ true intentions, we implemented a consistency check through strategically placed lie detector questions (Xu, 2011). Three questions sharing the same stem but with differently ordered response options were distributed throughout the questionnaire. This design enabled us to identify and subsequently exclude 9 invalid questionnaires that showed inconsistent responses across these verification items. A total of 265 questionnaires were initially distributed, and 247 valid consecutive questionnaires were recovered at the completion of the three time points, representing an effective recovery rate of 93.21%. In order to minimize attrition rates and sustain participant motivation throughout the longitudinal investigation, the study implemented a lottery-based incentive system delivering rewards between 5 and 20 RMB via the WeChat survey interface following questionnaire completion.

### Statistical methods

The correlation statistical analysis was conducted using the SPSS 26.0 and Mplus 8.0 software. Normally distributed measurement data were presented as mean ± standard deviation, while non-normally distributed data were expressed as median (interquartile range). Categorical variables were reported as frequencies and percentages. Pearson correlation analysis was performed for normally distributed variables, whereas Spearman correlation analysis was used for non-normally distributed variables. This study adopts the Cross-Lagged Panel Model (CLPM) and the latent variable Growth Model (LGM), aiming to simultaneously capture the dynamic interaction and individual development trajectory of dyadic coping and subjective well-being. CLPM was chosen because it can control the stability of the variable itself through an autoregressive path and test the temporal prediction effect between dyadic coping and subjective well-being through a cross-lag path, meeting the temporal sequence requirements of causal inference. LGM describes the individual growth trend through latent variables such as intercept (initial level) and slope (speed of change), and tests the differences among individuals. To control confounding factors, the comprehensive model captures the covariance of time-invariant confounding variables (such as gender and baseline characteristics) through random interceptions of intra-individual effects, and further controls time-varying confounding through instrumental or covariate adjustments. This method combination requires at least three time points to identify the growth curve and select the optimal model through model comparison (such as BIC), taking into account both dynamic relationships and the robustness of long-term trends. The model fit was assessed using multiple criteria (Browne and Cudeck, 1992): (1) χ^2^/df < 5; (2) Comparative Fit Index (CFI) > 0.90; (3) Tucker-Lewis Index (TLI) > 0.90; (4) Root Mean Square Error of Approximation (RMSEA) < 0.08; and (5) Standardized Root Mean Square Residual (SRMR) < 0.08. These thresholds collectively indicate good model fit. Missing data were imputed using multiple imputation methods, with sensitivity analyses demonstrating stable results after imputation (path coefficient fluctuations <10%). The Little’s MCAR test was conducted to assess the missing data mechanism (χ^2^ = 32.15, *p* = 0.12), confirming that the missing values were completely at random (MCAR). Multiple imputation was performed using the R language mice package (imputation cycles = 20, predictive mean matching method), with the root mean squared difference (RMSD) between the imputed and original data correlation matrices being 0.03, indicating minimal discrepancy (<0.05).

This study tested four core hypotheses: (1) dyadic coping and subjective well-being demonstrate synchronous growth trajectories (supporting H1); (2) dyadic coping serves as a stronger predictor of subjective well-being than vice versa (effect size H2 > H3); (3) their bivariate growth trajectories exhibit reciprocal predictive relationships (supporting H4). These findings collectively validate the “coping-well being” bidirectional enhancement model.

## Results

### General demographic information

**Figure 1:**
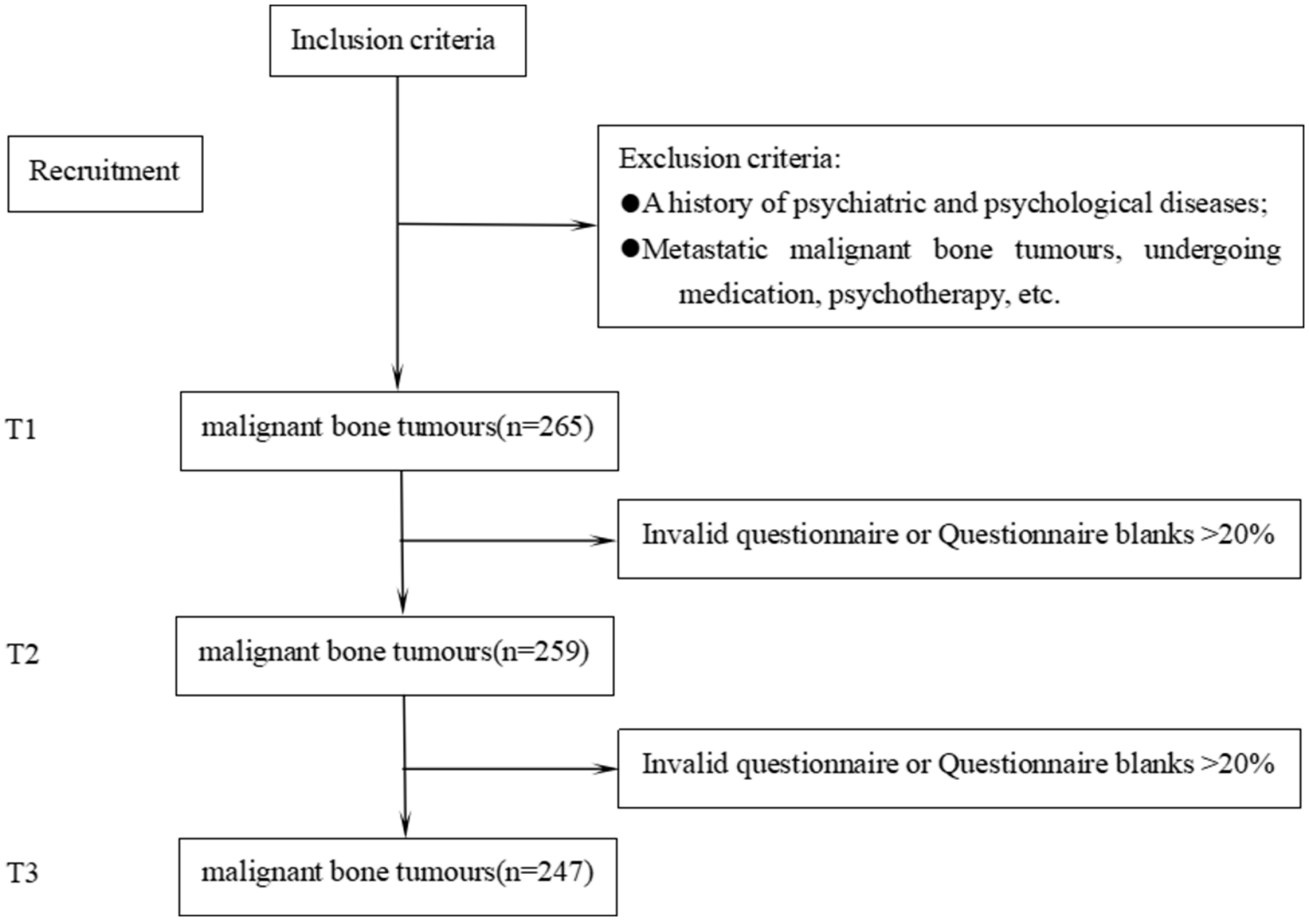
Participant flow chart.

A total of 247 valid questionnaires were collected for analysis in this study. Of these, 161 (65.2%) were completed by males and 86 (34.8%) by females. The participant flowchart is shown in Figure 1. The general information is presented in Table 1.

**Table 1:** General demographic information of patients with bone tumors (*n* = 247).

Items	Classifications	Number	(%)
Age (years)	<45	52	21.1
	45–59	137	55.5
	≥60	58	23.4
Gender	Male	161	65.2
	Female	86	34.8
Residence	City	76	30.8
	Towns	67	27.1
	Rural	104	42.1
Monthly family income (yuan)	<4,000	62	25.1
	4,000–6,000	96	38.9
	6,001–10,000	66	26.7
	>10,000	23	9.3
Educational level	Primary and below	52	21.1
	Junior high school	86	34.7
	Senior high school	75	30.4
	Colleges and above	34	13.8
Residency style	Live alone	28	11.3
	Family living	219	88.7

The objective of this study was to examine the relationship between dyadic coping and subjective well-being in patients with malignant bone tumors at three distinct time points (Table 2).

**Table 2:** Correlation analysis between dyadic coping and subjective well-being at 3 time points in patients with malignant bone tumors (*r* value, *n* = 247).

Items	*M*	*SD*	①	②	③	④	⑤	⑥
①dyadic coping T1	112.6	26.7	1					
②dyadic coping T2	115.7	26.5	0.724**	1				
③dyadic coping T3	116.7	24.5	0.624**	0.694**	1			
④well-being T1	64.8	15.6	0.361**	0.377**	0.415**	1		
⑤well-being T2	67.8	15.2	0.425**	0.435**	0.433**	0.761**	1	
⑥well-being T3	71.1	14.3	0.335**	0.415**	0.417**	0.526**	0.569**	1

**Significant correlation at the 0.01 level (two-tailed).

The objective of this study is to examine the relationship between dyadic coping and subjective well-being in patients with malignant bone tumors using a cross-lagged model.

**Figure 2:**
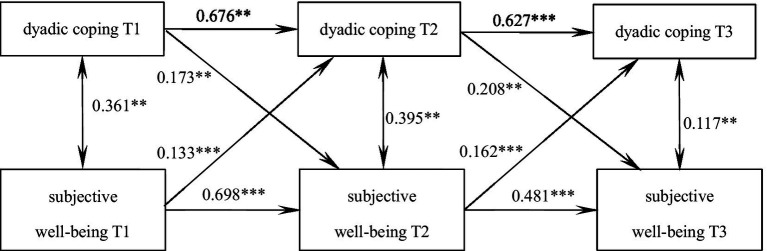
The predictive pathways of dyadic coping and subjective well-being in patients with malignant bone tumors at three time points. Control the baseline scores of age, gender and tumor stage; *** indicates *p* < 0.001, ** indicates *p* < 0.01.

The cross-lagged model was developed for the purpose of examining the inter-predictive relationship between dyadic coping and subjective well-being. The model demonstrated an excellent fit, with a χ^2^/df = 2.070, GFI = 0.972, TLI = 0.922, RMSEA = 0.128. As illustrated in Figure 1, dyadic coping and subjective well-being exhibited mutual predictive relationships at the initial level. Furthermore, the level of dyadic coping, on average, demonstrated a significant and positive predictive effect on the subsequent node of subjective well-being. Similarly, the level of subjective well-being exhibited a significant and positive predictive effect on the subsequent node of dyadic coping. The specific paths are depicted in Figure 2.

### Parallel latent variables of dyadic coping and subjective well-being in patients with malignant bone tumors

#### The course of dyadic coping in patients with malignant bone tumors

**Figure 3:**
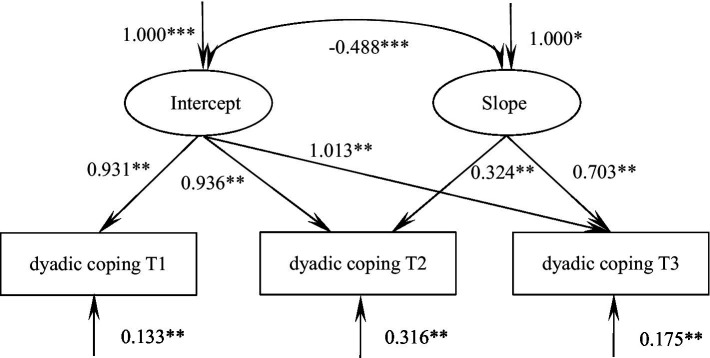
Model of dyadic coping for patients with malignant bone tumors. The values on the arrows pointing toward dyadic coping represent mean residuals. *** indicates *p* < 0.001, ** indicates *p* < 0.01, and * indicates *p* < 0.05.

The unconditional latent variable linear growth model based on dyadic coping in patients with malignant bone tumors was fitted with the following goodness of fit indicators: χ^2^/df = 0.084, GFI = 1.000, TLI = 1.008, RMSEA = 0.000, SRMR = 0.003. The model intercept, i.e., the initial value of dyadic coping of 112.60, showed an increasing trend in the subsequent three measurements [slope(S) = 0.228, *p* = 0.047], and there was a significant correlation between the intercept and the slope (*r* = −0.488, *p* < 0.001), suggesting that there was a significant negative correlation between the initial state of dyadic coping in patients with malignant bone tumors and the speed of development, i.e., the higher the initial level of dyadic coping in patients with malignant bone tumors, the slower the rate of increase in the later stage (Figure 3).

#### Trajectories of subjective well-being in patients with malignant bone tumors

**Figure 4:**
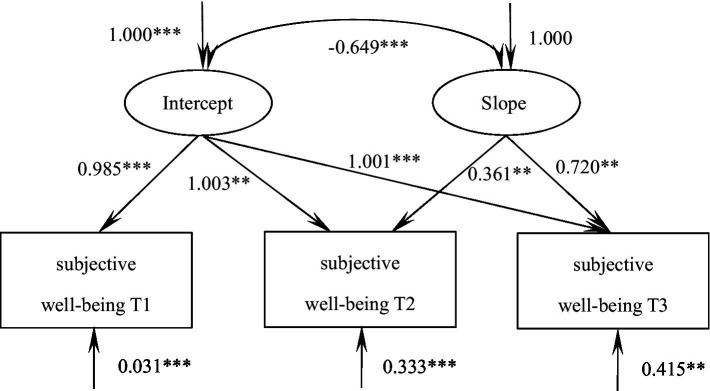
Model of subjective well-being for patients with malignant bone tumors. The values on the arrows pointing toward subjective well-being represent mean residuals. *** indicates *p* < 0.001, ** indicates *p* < 0.01.

According to the unconditional latent variable linear growth model of subjective well-being in patients with malignant bone tumors, the goodness of fit indicators were as follows χ^2^/df = 7.786, GFI = 0.978, TLI = 0.935, RMSEA = 0.166, SRMR = 0.032, which was a good fit. The intercept of the model, i.e., the initial value of subjective well-being 64.77, showed an increasing trend in the following three measurements (S = 0.109, *p* = 0.212), and there was a significant correlation between the intercept and the slope (*r* = −0.649, *p* < 0.001), suggesting that there was a significant negative correlation between the initial state of subjective well-being and the rate of development in patients with malignant bone tumors, i.e., The higher the initial level, the slower the rate of increase in the later stages, see Figure 4.

#### Dynamic relationship between dyadic coping and subjective well-being in patients with malignant bone tumors

**Figure 5:**
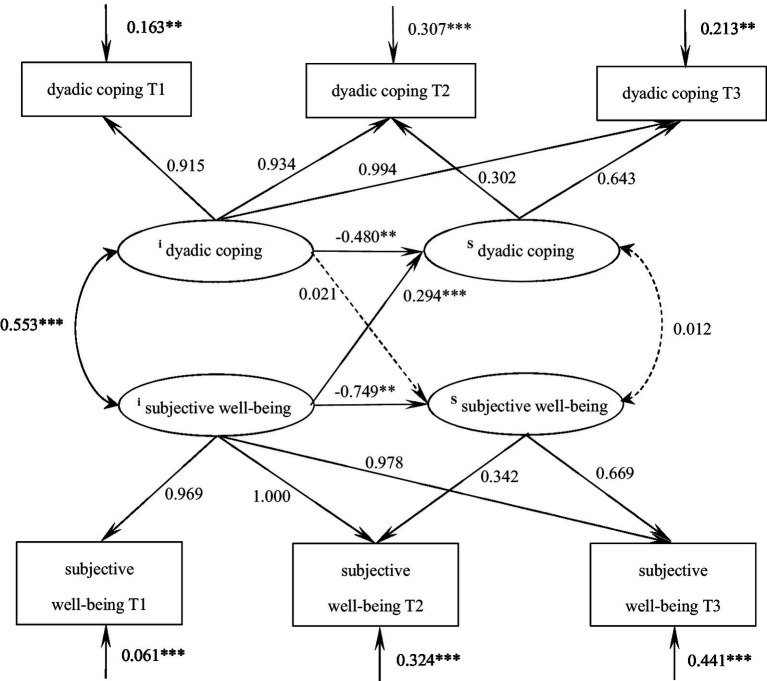
Parallel latent variable model of dyadic coping and subjective well-being in patients with malignant bone tumors. The values on the arrows pointing toward dyadic coping/subjective well-being represent mean residuals. *** indicates *p* < 0.001, ** indicates *p* < 0.01.

The parallel latent variable growth model of dyadic coping and subjective well-being in patients with malignant bone tumors was constructed and the goodness of fit indices were as follows: χ^2^/df = 2.048, GFI = 0.990, TLI = 0.979, RMSEA = 0.065, SRMR = 0.036, which was a good fit. At the baseline level, dyadic coping demonstrated a significant positive correlation with subjective well-being (*r* = 0.533, *p* < 0.001), indicating that patients with higher initial dyadic coping levels exhibited greater well-being. The latent growth model further revealed that the baseline level of dyadic coping negatively predicted its subsequent growth rate (*β* = −0.480, *p* = 0.008), suggesting a deceleration effect for those with initially high coping skills. Similarly, the baseline subjective well-being level negatively influenced its own developmental trajectory (*β* = −0.749, *p* = 0.005), while paradoxically, it positively predicted the growth rate of dyadic coping (*β* = 0.294, *p* < 0.001), implying that lower initial well-being was associated with slower progress in coping skills development. These dynamic relationships are visually summarized in Figure 5.

## Discussion

### Relationship between dyadic coping and subjective well-being in patients with malignant bone tumors

By constructing a cross-lagged model, this study found that, on average, dyadic coping levels significantly and positively predicted subjective well-being at the next node, and subjective well-being levels similarly significantly and positively predicted dyadic coping levels at the next node. Previous studies have shown that the subjective well-being of cancer patients is at a low to moderate level and is closely related to the presence of symptom burden, depression status, and quality of survival (Lameiras et al., 2018; Ryu et al., 2022). Patients with malignant bone tumors demonstrated moderate levels of subjective well-being, showing some discrepancies with prior research findings (Lameiras et al., 2018; Ryu et al., 2022). Patients with malignant bone tumors demonstrated moderate levels of subjective well-being, showing notable differences from findings in previous studies. These discrepancies may be attributed to several factors, including variations in demographic characteristics of the study populations and differences in measurement instruments. For instance, Ryu et al.’s study (Ryu et al., 2022) focused exclusively on female breast cancer patients and employed simplified well-being assessment tools (this study adopts the General Well-Being Schedule)—methodological differences that could significantly influence the measurement of subjective well-being. Many factors such as temperament, income, supportive social relationships, cultures, health and longevity are all related to subjective well-being (Diener et al., 2018). Previous studies have described the cross-sectional relationship between dyadic coping and subjective well-being, and the present study confirms that the level of dyadic coping in patients with bone tumors predicts the level of well-being in the next stage, which may be attributed to the fact that patients with higher levels of dyadic coping tend to receive more psychological support and life care from their spouses after the disease, and that negative emotions generated after the disease can be effectively exported and released (Chen et al., 2021). Spouse as the primary caregiver, effective communication can alleviate the negative emotions generated by the patient, so that the patient to maintain a more stable state of mind (Wolff et al., 2020). In addition, the quality of life of patients can be largely maintained by the life care of their spouses, so that their subjective well-being level in the next stage is higher. Similarly, subjective well-being in bone tumor patients positively predicts subsequent dyadic coping levels. Enhanced psychological resilience, elevated hope, and improved positive emotion regulation—facilitated by higher well-being—strengthen couples’ communication efficacy and collaborative coping strategies. This accumulation of psychological resources not only buffers disease-related stress but also fosters a reciprocal cycle of mutual support, thereby improving future dyadic coping outcomes. This suggests that clinical staff should pay attention to the assessment of dyadic coping and subjective well-being levels of bone tumor patients, take advantage of the existence of an interactive relationship between the two, encourage patients to communicate with their spouses, and at the same time reduce the reduction of patients’ disease-related quality of life, and have a positive effect on the improvement of well-being in the later stage.

### Dynamics of dyadic coping and subjective well-being in patients with malignant bone tumors

In this study, we found that patients with malignant bone tumors showed an increasing trend in dyadic coping and subjective well-being after diagnosis using a latent variable growth model. Most previous longitudinal studies of dyadic coping have been limited to malignant tumors, and the trend of change in dyadic coping is controversial. Vaske et al. (2015) pointed out that the level of dyadic coping showed a trend of decreasing in 3 years, the study of Ernst et al. (2017) showed that the level of dyadic coping of patients did not have a significant change in 6 months of their illness, and the study of Shi (2021) supported the fact that the level of dyadic coping of patients undergoing chemotherapy for malignant tumors also showed a trend of increasing and then decreasing. The trend of the level of dyadic coping varies according to the type of disease, patient demographics and the instrument used for measurement in the included population. The longitudinal selection time of this study was relatively short, the patients had more negative emotions in the early stage of the disease, and the spouses faced with the sudden increase in the burden of care of the patient’s illness, the dyadic coping was at a lower level (Zimmermann et al., 2021). With the treatment and recovery of the disease, the spouse through effective communication with the patient, the bad mood is effectively cathartic, dyadic coping, well-being showed an upward trend.

The results of the parallel latent variable growth model showed a positive correlation between dyadic coping and well-being at the initial level, i.e., the higher the patient’s level of dyadic coping, the higher his or her level of subjective well-being. This has been confirmed in previous studies (Chen et al., 2022): at the onset of the disease, the higher the level of support patients received from their spouses, the lower the output of negative emotions and the life care provided by their spouses, the higher the level of subjective well-being of the patients. Higher initial levels of dyadic coping and subjective well-being demonstrate a negative predictive effect on subsequent developmental trajectories, with elevated baseline scores correlating with slower growth rates. This phenomenon can be explained through the lens of emotional adaptation asymmetry, whereby individuals with superior initial adaptation capacities exhibit accelerated habituation to positive stimuli, consequently constraining potential for further enhancement and producing diminishing marginal returns in positive affect (Li et al., 2015). These mechanisms collectively account for the observed inverse relationship between initial functioning levels and subsequent developmental velocity. Therefore, the higher the initial level of dyadic coping and subjective well-being, the slower the increase. The initial level of subjective well-being positively predicts the development of dyadic coping, i.e., the lower the initial level of subjective well-being, the slower the increase in dyadic coping. Patients with lower levels of subjective well-being at the onset of the disease tend to have accumulated negative emotions and a poor quality of life, and these patients tend to avoid facing their own illness and communicate less with their spouses, so the level of dyadic coping rises more slowly.

### Shortcomings and prospects

This longitudinal study examined the changing trajectories of dyadic coping and subjective well-being in 247 patients diagnosed with malignant bone tumors during their first 3 months post-diagnosis, providing valuable insights for clinical monitoring of these psychological changes. The findings highlight the importance of prioritizing clinical interventions for patients exhibiting lower baseline subjective well-being. Implementing spouse-involved dual-system support interventions—including joint psychological counseling and collaborative problem-solving training—can effectively address avoidance behaviors in low well-being patients and enhance their dyadic coping capacities. Crucially, early assessment of patients’ emotional baselines followed by tailored intervention strategies represents the most effective approach for achieving optimal long-term therapeutic outcomes.

This study has several methodological limitations that warrant careful consideration. The use of convenience sampling may introduce selection bias and reduce sample heterogeneity, potentially limiting the generalizability of findings to broader populations (Hu, 2014). Furthermore, the limited number of longitudinal assessment timepoints may obscure nonlinear trajectories in variable changes, consequently diminishing statistical power to detect meaningful temporal patterns (Yang et al., 2017). Additionally, reliance on patient-reported outcome measures carries inherent risks of response bias, as participants may consciously or unconsciously modify their responses, potentially compromising data validity and introducing measurement bias (Yang et al., 2017). These limitations should be acknowledged when interpreting the study results and considering their clinical implications.

While the second-order approach theoretically offers advantages in disentangling measurement error from true trajectory variation—thereby providing more precise estimates of growth parameters (e.g., intercept and slope variances)—its implementation necessitates larger sample sizes and more complex model specifications. Given the current study’s sample size constraints and the prioritization of analytical parsimony, we adopted the first-order method as a pragmatic alternative. This methodological choice inherently limits the precision of trajectory estimation, particularly in accounting for measurement error attenuation effects, and thus warrants cautious interpretation of the reported growth parameters. Future research with adequate statistical power should prioritize second-order latent growth modeling to enhance the accuracy of individual change trajectory estimation and better capture the hierarchical structure of longitudinal data.

## Conclusion

Dyadic coping and subjective well-being of malignant bone tumor patients showed an upward trend, and dyadic coping and subjective well-being had a mutual predictive effect, indicating that dyadic coping and subjective well-being of malignant bone tumor patients were closely related. The initial level of dyadic coping and subjective well-being is negatively predictive of their own development speed, the initial level of dyadic coping can positively predict the development speed of subjective well-being, and the initial level of subjective well-being can positively predict the development speed of dyadic coping, so we pay attention to the assessment of dyadic coping level of patients with malignant bone tumors in different periods, and encourage the patients’ family members to carry out effective communication to improve the level of dyadic coping. In addition, it is beneficial to improve the subjective well-being of patients with malignant bone tumors, which in turn reduces psychological distress and improves prognosis.

## What is already known

- Malignant bone tumors lead to physical disability, severely affecting the quality of survival, with extremely high levels of psychological distress and low levels of subjective well-being among patients.
- Low levels of subjective well-being result in the accumulation of negative emotions, reduced treatment compliance, and even misanthropy in patients with bone tumors.
- Good dyadic coping can alleviate the adverse emotions present in patients, improve their cooperation and quality of life, and improve their prognosis.

## What this paper adds

- Dyadic coping and subjective well-being in bone tumor patients 6 months after diagnosis all show upward trajectories.
- Dyadic coping levels in bone tumor patients on average significantly and positively predicted subjective well-being at the next node, and subjective well-being levels similarly significantly and positively predicted dyadic coping levels at the next node.
- Initial levels of subjective well-being and rate of development in bone tumor patients negatively predict each other, and initial levels of subjective well-being positively predict the rate of development of dyadic coping.

## Data Availability

The raw data supporting the conclusions of this article will be made available by the authors, without undue reservation.
